# Supplementary material for: End Sequence Analysis Toolkit (ESAT) expands the extractable information from single-cell RNA-seq data
Source: Genome Res. 2016 Oct;26(10):1397–410. doi: 10.1101/gr.207902.116 (PMC5052061; doi:10.1101/gr.207902.116)
Supplement: Supplemental Material [file supp_gr.207902.116_Supp_Materials.tar.gz › ESAT-esat_v0.1/doc/html/classbroad_1_1pda_1_1geneexpression_1_1_score_diff_genes.html]

ESAT: broad.pda.geneexpression.ScoreDiffGenes Class Reference


ESAT


- Main Page
- Related Pages
- Packages
- Classes
- Files

- Class List
- Class Index
- Class Hierarchy
- Class Members

All Classes Namespaces Files Functions Variables Enumerator Pages

- broad
- pda
- geneexpression
- ScoreDiffGenes

Static Public Member Functions |
List of all members

broad.pda.geneexpression.ScoreDiffGenes Class Reference

|  |  |
| --- | --- |
| Static Public Member Functions | |
| static MatrixWithHeaders | scoreListByTStatistic (MatrixWithHeaders expression, Map< String, ArrayList > groupIndexes, String controlClass, double fudgeFactor) |
|  | |
| static ArrayList | getREST (String group1, Map< String, ArrayList > groupIndexes) |
|  | |

## Member Function Documentation

|  |  |  |  |
| --- | --- | --- | --- |
| static ArrayList broad.pda.geneexpression.ScoreDiffGenes.getREST | ( | String | *group1*, |
|  |  | Map< String, ArrayList > | *groupIndexes* |
|  | ) |  |  |

static

Here is the caller graph for this function:

|  |  |  |  |
| --- | --- | --- | --- |
| static MatrixWithHeaders broad.pda.geneexpression.ScoreDiffGenes.scoreListByTStatistic | ( | MatrixWithHeaders | *expression*, |
|  |  | Map< String, ArrayList > | *groupIndexes*, |
|  |  | String | *controlClass*, |
|  |  | double | *fudgeFactor* |
|  | ) |  |  |

static

Here is the call graph for this function:

---

The documentation for this class was generated from the following file:

- java/broad/pda/geneexpression/ScoreDiffGenes.java


---

Generated on Fri Aug 15 2014 15:11:00 for ESAT by  

 1.8.7
